# Flood syndrome following right-sided heart failure: a case report

**DOI:** 10.1093/jscr/rjab631

**Published:** 2022-01-25

**Authors:** Marko Murruste, Karri Kase, Martin Kivilo, Urmas Lepner

## Abstract

Flood syndrome is a rare condition, where a patient with ascites suffers sudden umbilical hernia rupture and a subsequent drainage of ascitic fluid from the abdominal cavity. The cause of ascites is usually liver cirrhosis. Complications associated with cirrhosis of the liver make Flood syndrome difficult to manage. In these cases, conservative management has been associated with high morbidity. We describe, to our knowledge, for the first time a patient with Flood syndrome caused by right-sided heart failure. We also show that conservative treatment gives great initial outcome and is a viable option for this type of Flood syndrome.

## INTRODUCTION

Flood syndrome is a condition where a patient with ascites and an umbilical hernia suffers a sudden rupture of the hernia and a subsequent drainage of ascitic fluid from the abdominal cavity [1]. Optimal management of this rare case is still a matter of debate [2–4]. The cause of ascites in Flood syndrome is almost always liver cirrhosis [5]. In this case report we provide insight into our experience with a rare case of Flood syndrome following right-sided heart failure and present a rare video of umbilical hernial leakage.

## CASE REPORT

A 59-year-old male was taken to the emergency department due to the rupture of umbilical hernia. The leakage of fluid started after the eschar covering the patient’s hernia came off. The patient presented with multiple comorbidities: type II diabetes, hypertension, kidney failure, right-sided heart failure (NYHA class IV) and pulmonary artery hypertension. Right-sided heart failure was caused by repeated episodes of pulmonary artery thrombosis. When heart failure decompensated 1 year earlier, ascites and oedema first developed. The patient had been mostly bedridden since. It is noteworthy that he took his medications infrequently and sporadically. Ascites led to an evident umbilical hernia. Although there was no liver cirrhosis, steatosis had been noted previously. This can be attributed to occasional episodes of alcohol abuse.

At presentation the patient had a massive abdominal distension and anasarca. Fluid leakage from the umbilical hernia was visible (see the provided video). There was no serious electrolyte imbalance (sodium 135 mmol/L, potassium 4.7 mmol/L, chloride 1.13 mmol/L). Albumin was 41 g/L and total protein was 70 g/L. The patient’s kidney function had deteriorated further with an eGFR (estimated glomerular filtration rate) of 22 ml/min/1.73m^2^ and urea of 21.6 mmol/L. In accordance with the massive heart failure, NT-proBNP (N-terminal pro-brain natriuretic peptide) was 4907 ng/L. There was no indication of liver damage: alanine aminotransferase 17 U/L and aspartate aminotransferase 30 U/L. The patient was hepatitis C-virus and hepatitis-B virus negative.

Hernial leakage was collected into an ostomy bag and an ascites drain was placed in the left side of the abdomen. A total of 11 L of the ascites fluid were removed during the first day and further 2 L, during the following few days. The leakage site was covered with a sterile dressing, no surgical fixation of the hernia was undertaken. Furthermore, no antibiotic therapy was needed as the C-reactive protein values never rose above 23 mg/L. The patient’s heart failure medications were adjusted and, with advice to adhere to the treatment regimen, the patient left the hospital after 9 days.

Five months later the patient was rehospitalized. This time the hernia was incarcerated with small bowel strangulation. There was no evidence of ascites. Immediate surgery was undertaken, and a viable bowel loop was released from the hernial sac. The hernia was suture repaired. The postoperative period was uneventful, and the patient was discharged from the hospital 7 days after surgery. As of November 2021, the patient is alive and there have occured no further complications regarding the hernia.

## DISCUSSION

As many as 20% of cirrhotic patients with ascites develop an umbilical hernia. The elevated intraabdominal pressure caused by ascites leads to umbilical herniation; further compression against the skin causes its necrosis and thinning, which leads to eventual rupture. Thus, Flood syndrome is established [1, 5]. We found no previous record of Flood syndrome following ascites, caused by right-sided heart failure. In previous case reports and reviews the predominant cause of ascites has been cirrhosis of the liver. Despite the difference in the underlying causes, both cirrhosis and right-sided heart failure can lead to ascites. As the latter is the driver for development of Flood syndrome, somewhat similar strategies in the management of Flood syndrome should apply.

As Flood syndrome is rare, there exist only a few case reviews. And, as mentioned, they focus on cases with liver cirrhosis. In a review of 30 patients with cirrhosis and Flood syndrome, the average age of the patients was 51 years and 73% of the patients were men [6]. This might not apply to our case, as the indicators above are affected by the epidemiology of the underlying disease. Ulceration of the skin over the hernia precedes a rupture in 73% of cases. The rupture of hernia usually occurs spontaneously. In a few cases local trauma or sudden elevation of intraabdominal pressure causes the leakage. No cardiovascular deterioration normally ensues [6]. Our experience was no different: loosening of an eschar led to the initial massive leakage of ascites fluid, without cardiovascular deterioration.

The most common complications of Flood syndrome are fluid imbalance, infection and bowel evisceration/incarceration [5]. No infection, serious fluid imbalance or immediate bowel incarceration was seen in our patient. We assume that the rate of complications should be higher in patients with Flood syndrome stemming from cirrhosis.

Optimal treatment of Flood syndrome is still under debate. In a general sense, it should be surgical, as mortality in the case of conservative treatment alone can be as high as 60–80%. However, urgent surgical repair is associated with higher rate of complications. Before surgical management, the underlying condition should be corrected, to reduce complications and recurrence. Firstly, the amount of peritoneal fluid leakage must be controlled and the wound should be cleaned. An ostomy bag can be used to collect further ascites fluid. Secondly, management of ascites is needed to prevent later wound dehiscence and evisceration [5]. These recommendations were followed in the present case, despite its different essential pathology. However, in our case, we saw good initial response to the conservative management. Yet, we believe that surgical treatment of Flood syndrome following right-sided heart failure is also a safe option, as cirrhosis brings with it coagulopathy and thrombocytopenia. Primary closure with non-absorbable sutures is the surgical method of choice, as mesh hernioplasty is associated with higher risk of infection [2, 5]. In our case surgery was needed due to the later occurrence of strangulated hernia; the hernia was suture repaired.

In conclusion, Flood syndrome following right-sided heart failure can initially be managed successfully conservatively, although this can require later intervention.

## Supplementary Material

Flood_syndrome_rjab631

## References

[1] Flood FB . Spontaneous perforation of the umbilicus in Laënnec’s cirrhosis with massive ascites. N Engl J Med 1961;264:72–4. 13700320 10.1056/NEJM196101122640204

[2] Chatzizacharias NA, Bradley JA, Harper S, et al. Successful surgical management of ruptured umbilical hernias in cirrhotic patients. World J Gastroenterol 2015;21:3109–13. 25780312 10.3748/wjg.v21.i10.3109 PMC4356934

[3] Smith MT, Rase B, Woods A, et al. Risk of hernia incarceration following transjugular intrahepatic portosystemic shunt placement. J Vasc Interv Radiol 2014;25:58–62. 24269791 10.1016/j.jvir.2013.09.003

[4] Chikamori F, Mizobuchi K, Ueta K, et al. Flood syndrome managed by partial splenic embolization and percutaneous peritoneal drainage. Radiol Case Reports 2021;16:108–12. 10.1016/j.radcr.2020.10.045 PMC7649598 33204382

[5] Strainiene S, Peciulyte M, Strainys T, et al. Management of Flood syndrome: what can we do better? World J Gastroenterol 2021;27:5297. 34539133 10.3748/wjg.v27.i32.5297 PMC8409160

[6] Lemmer JH, Strodel WE, Knol JA, Eckhauser FE. Management of spontaneous umbilical hernia disruption in the cirrhotic patient. Ann Surg 1983;198:30. 6859990 10.1097/00000658-198307000-00006 PMC1352927

